# Genetic Variants and Protective Immunity against SARS-CoV-2

**DOI:** 10.3390/genes13122355

**Published:** 2022-12-13

**Authors:** Ali A. Rabaan, Abbas Al Mutair, Mohammed Aljeldah, Basim R. Al Shammari, Tarek Sulaiman, Abeer N. Alshukairi, Mubarak Alfaresi, Jumana M. Al-Jishi, Neda A. Al Bati, Maha A. Al-Mozaini, Ali Al Bshabshe, Jenan A. Almatouq, Abdulmonem A. Abuzaid, Amal H. Alfaraj, Wasl Al-Adsani, Mohammed Alabdullah, Sara Alwarthan, Fatimah Alsalman, Ameen S. S. Alwashmi, Saad Alhumaid

**Affiliations:** 1 Molecular Diagnostic Laboratory, Johns Hopkins Aramco Healthcare, Dhahran 31311, Saudi Arabia; 2 College of Medicine, Alfaisal University, Riyadh 11533, Saudi Arabia; 3 Department of Public Health and Nutrition, The University of Haripur, Haripur 22610, Pakistan; 4 Research Center, Almoosa Specialist Hospital, Al-Ahsa 36342, Saudi Arabia; 5 College of Nursing, Princess Norah Bint Abdulrahman University, Riyadh 11564, Saudi Arabia; 6 School of Nursing, Wollongong University, Wollongong, NSW 2522, Australia; 7 Nursing Department, Prince Sultan Military College of Health Sciences, Dhahran 33048, Saudi Arabia; 8 Department of Clinical Laboratory Sciences, College of Applied Medical Sciences, University of Hafr Al Batin, Hafr Al Batin 39831, Saudi Arabia; 9 Infectious Diseases Section, Medical Specialties Department, King Fahad Medical City, Riyadh 12231, Saudi Arabia; 10 Department of Medicine, King Faisal Specialist Hospital and Research Center, Jeddah 21499, Saudi Arabia; 11 Department of Pathology and Laboratory Medicine, Sheikh Khalifa General Hospital, Umm Al Quwain 499, United Arab Emirates; 12 Department of Pathology, College of Medicine, Mohammed Bin Rashid University of Medicine and Health Sciences, Dubai 505055, United Arab Emirates; 13 Internal Medicine Department, Qatif Central Hospital, Qatif 35342, Saudi Arabia; 14 Medical and Clinical Affairs, Rural Health Network, Eastern Health Cluster, Dammam 31444, Saudi Arabia; 15 Immunocompromised Host Research Section, Department of Infection and Immunity, King Faisal, Specialist Hospital and Research Centre, Riyadh 11564, Saudi Arabia; 16 Adult Critical Care Department of Medicine, Division of Adult Critical Care, College of Medicine, King Khalid University, Abha 62561, Saudi Arabia; 17 Department of Clinical Laboratory Sciences, Mohammed Al-Mana College of Health Sciences, Dammam 34222, Saudi Arabia; 18 Medical Microbiology Department, Security Forces Hospital Programme, Dammam 32314, Saudi Arabia; 19 Pediatric Department, Abqaiq General Hospital, First Eastern Health Cluster, Abqaiq 33261, Saudi Arabia; 20 Department of Medicine, Infectious Diseases Hospital, Kuwait City 63537, Kuwait; 21 Department of Infectious Diseases, Hampton Veterans Administration Medical Center, Hampton, VA 23667, USA; 22 Department of Infectious Diseases, Almoosa Specialist Hospital, Al Mubarraz 36342, Saudi Arabia; 23 Department of Internal Medicine, College of Medicine, Imam Abdulrahman Bin Faisal University, Dammam 34212, Saudi Arabia; 24 Department of Emergency Medicine, Oyun City Hospital, Al-Ahsa 36312, Saudi Arabia; 25 Department of Medical Laboratories, College of Applied Medical Sciences, Qassim University, Buraydah 51452, Saudi Arabia; 26 Administration of Pharmaceutical Care, Al-Ahsa Health Cluster, Ministry of Health, Al-Ahsa 31982, Saudi Arabia

**Keywords:** coronaviruses, COVID-19, Genetic resistance, protective immunity

## Abstract

The novel coronavirus-19 (SARS-CoV-2), has infected numerous individuals worldwide, resulting in millions of fatalities. The pandemic spread with high mortality rates in multiple waves, leaving others with moderate to severe symptoms. Co-morbidity variables, including hypertension, diabetes, and immunosuppression, have exacerbated the severity of COVID-19. In addition, numerous efforts have been made to comprehend the pathogenic and host variables that contribute to COVID-19 susceptibility and pathogenesis. One of these endeavours is understanding the host genetic factors predisposing an individual to COVID-19. Genome-Wide Association Studies (GWAS) have demonstrated the host predisposition factors in different populations. These factors are involved in the appropriate immune response, their imbalance influences susceptibility or resistance to viral infection. This review investigated the host genetic components implicated at the various stages of viral pathogenesis, including viral entry, pathophysiological alterations, and immunological responses. In addition, the recent and most updated genetic variations associated with multiple host factors affecting COVID-19 pathogenesis are described in the study.

## 1. Introduction

Since COVID-19 was initially characterised in December 2019 [1,2], our knowledge about the mechanistic aspects of this life-threatening disease has significantly developed. Researchers are still trying to understand the genetic foundation of innate human immunity to SARS-CoV-2. The infection rate of SARS-CoV-2 in certain houses can range up to 70% [3,4], and a few households have been recorded in which all residents are sick except one of the family members [5], indicating that people with prolonged exposure to SARS-CoV-2 may display resistance to virus infection.

Specific Inborn Errors of Immunity (IEIs) have determined vulnerability towards acute COVID-19 since the outbreak of the COVID-19 pandemic. The COVID-19 Human Genomic Effort found IEIs to span eight genomic loci controlling the induction of type I Interferon (IFN) via TLR3 and IRF7 in 23 critically sick individuals [6]. Four unrelated and formerly healthy individuals observed autosomal and recessive mutations in the genetic loci encoding IRF7 or IFNAR1, leading to its deficiency. People with IEIs reveal that a type I IFN immune response is required to control COVID-19 infection. This observation led to the finding of already existing neutralising autoantibodies to type I IFNs, which denote IEIs related to type I IFN [1]. According to subsequent research in independent cohorts, over 10% of people with severe COVID-19 had neutralising autoantibodies against type I IFNs. According to a consortium, autoantibodies balancing the physiological amounts of type I IFNs are present in around 20% of individuals above 70 years-old, and with severe pneumonia [7]. Furthermore, a research consortium found that roughly 1% of male patients with severe pneumonia under 60 exhibited X-recessive TLR7 deficiency [8]. Before exposure to SARS-CoV-2, the people with IEIs and those with autoantibodies had no particular sensitivity to other severe viral illnesses.

This observation is in line with the fact that SARS-CoV-2 produces fewer type I IFNs than, say, the annual influenza virus [9]. However, one third of the adverse responses to the live attenuated yellow fever virus vaccination have been linked to type I IFN autoantibodies [10]. These cases show how the genetic explanation of an immune impairment in a few unusual individuals might reveal a system that is impaired in several people due to other factors. The number of people who display natural resistance to SARS-CoV-2 infection is unclear. Still, several lines of evidence have pointed to several candidate genes that may be implicated in the innate immune response that leads to the resistance towards SARS-CoV-2 infection. 

Genome-wide association studies (GWAS) were used to find the ABO gene [11,12]. Although early findings related to the influence of blood group on COVID-19 intensity displayed a diverse form, a recent meta-analysis involving approximately 50,000 participants from 46 studies indicated that this locus affected infection vulnerability. On the other hand, the O allele has a minor protective effect, with an odds ratio of only ~0.90. ABO blood types may also function as co-receptors for SARS-CoV-2, even though no unifying resistance mechanism has yet been proposed [11,13]. Pernio (chilblain) linked with the SARS-CoV-2 pandemic is an uncommon symptom in those who have been exposed to the virus, but it may provide knowledge about infection resistance mechanisms [14,15]. The skin lesions observed in familial chilblain lupus and Aicardi–Goutières syndrome, which are monogenic illnesses characterised by gene variations leading to an enhanced type I IFN response, are mimicked by pandemic-associated pernio (‘COVID toes’) [16]. Although most people with pernio are seronegative, skin biopsy specimens have shown the presence of the spike protein of SARS-CoV-2 and a robust local type I IFN response, suggesting early viral clearance [17]. These findings point to the existence of infection and, as a result, the lack of natural infection resistance. However, through a better understanding of this process, we may obtain an insight into the host mechanisms that limit viral replication and promote resistance to COVID-19 infection. 

Additional putative host genes that support the life cycle of the COVID-19 virus have been found by in vitro interactome analyses. COVID-19 infection requires the presence of an ACE2 receptor for entry into the host cell and the serine protease, TMPRSS2, for the priming of spike protein, which was found early in the pandemic [18,19,20,21]. Likewise, GWAS discovered an uncommon variation near ACE2 that is found to protect against SARS-CoV-2 infection, probably by lowering ACE2 expression [22]. Additionally, specific human ACE2 allelic variants bind the spike protein of SARS-CoV-2 with varying affinities, although their influence on infection is unclear [23]. In addition, a genome-wide CRISPR knockout screen identified TMEM41B as essential for SARS-CoV-2 and other coronaviruses infection [24]. Flaviviruses also need the endoplasmic reticulum transmembrane protein TMEM41B. Its influence on COVID-19 infection is unclear. However, an allele detected in East and South Asians was shown to cause reduced ability to sustain flavivirus infection [25].

Similarly, a combined affinity purification approach and mass spectrometry of human proteins associated with SARS-CoV-2 led to a massive protein-protein interaction map [26]. As a result of the functional study of this interactome, a list of important host variables for the transmission of COVID-19 was created [27]. Even though there isn’t any human research that links this interactome to the risk of infection, the genes involved and the loci found by GWAS can be looked at as possible places to find genetic variants that protect against infection. 

This review provides comprehensive and updated information about the genetic variations associated with host factors that impact the outcome of COVID-19. Although other reviews have covered such factors in brief, we have extended the discussion to include factors affecting various stages of viral pathogenesis and an updated list of associated mutations. Table 1 summarises the genetic factors associated with COVID-19 susceptibility. The keywords used to search for relevant data were ((genetic variant) OR (gene mutation)) AND ((resistance) OR (protection)) AND (immunity) AND ((SARSCOV-2) OR (corona virus) OR (covid) OR (SARSCOV2)) using the PubMed advanced search. These were last were searched on 30 June 2022. The majority of the data were collected from primary research articles published between 2019 and 2022 in order to present the most updated information about the mutations related to host genetic variables that influence the COVID-19 outcome.

## 2. Genetic Resistance to Virus Entry

Individuals with allelic variants of CCR5 that result in CCR5 deletion, termed “elite resistors” to the human immunodeficiency virus (HIV), are a suitable example of genetic resistance due to a lack of viral receptors on host cells. Interestingly, there is a predominance of this CCR5 mutation in Europeans. This might be influenced by the fact that it confers a reduced smallpox death rate [51,52,53,54]. It is unknown whether deletion of CCR5 precludes co-reception of the variola virus or lowers the viral-induced lethal immune response by inhibiting inflammatory chemokine signals. Moreover, acquiring mutations in the spike protein allowed it to bind with greater affinity to the human host cell receptors, ACE2 for SARS-CoV-2 and SARS-CoV or DPP4 for the Middle East respiratory syndrome-related coronavirus (MERS-CoV). This was a crucial step in the transition of coronaviruses from bats to humans [2]. 

**Figure 1:**
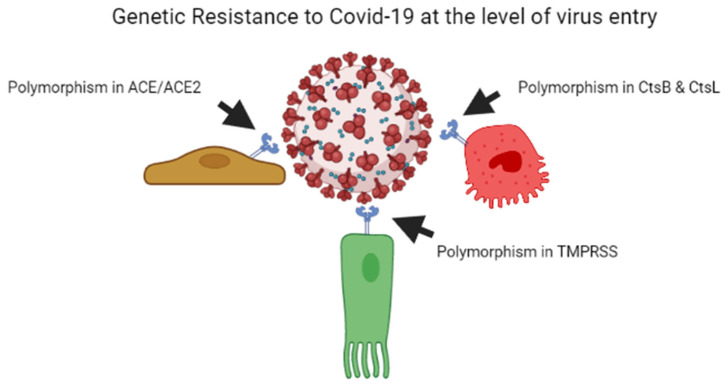
Genetic resistance of host against the COVID-19 virus at the level of entry of the virus.

As per previous studies, plant breeders recognised that genetic resistance to communicable illness is unstable over time; therefore, variant bacteria can be selected rapidly, escape resistance and spread better, whether through loss of receptors or by developing immunological responses [55,56]. Every year, the insecure resistance state is focused on preventing viral transmission. This is why we require a new seasonal influenza vaccination each year. Influenza elicits long-lasting neutralising antibodies, resulting in a stronger selection for the antigenic drift in virus epitopes, causing them to escape being recognised by the current set of antibodies. Coronaviruses possess the largest genomes of any RNA viruses, and, contrary to other RNA viruses, they copy genomes with a considerably greater degree of accuracy [57]. The COVID-19 virus develops point mutations at a slower rate of one in ten thousand bases yearly [58]. As a result, coronaviruses do not use antigenic drift as a technique to avoid neutralising antibody development, unlike influenza and human immunodeficiency virus vaccines. Instead, after human infection with the common cold coronavirus HCoV-229E [59,60] or severe conditions with SARS-CoV, neutralising antibody production is low and oddly brief [61,62]. In the poultry sector, attenuated vaccines against contagious coronavirus use are limited due to short-lived antibody production [63,64]. On the one hand, influenza evades the neutralising antibody yield due to a higher rate of mutations in the viral genome, while coronavirus alters the body’s mechanisms for neutralising antibody responses, leading to short-lived coronaviruses that do not halt viral spread in the population [60]. Figure 1 illustrates the mechanism of genetic resistance of the host against virus entry.

### 2.1. Angiotensin Converting Enzymes

Angiotensin I-converting enzyme (ACE) and angiotensin-converting enzyme-2 (ACE2) represent a pair of homologous genes that govern the physiological balance of the renin-angiotensin system (RAS). SARS-CoV-2 uses ACE2 receptors to infect susceptible cells [21]. In numerous methods, ACE and ACE2 influence each other’s expression levels [65]. Therefore, examining the genetic variations of these genes can assist us to determine why COVID-19 is more acute in certain individuals.

#### 2.1.1. Angiotensin-I Converting Enzyme

The insertion or deletion of a 287 bp Alu repeat is a frequent polymorphism of the ACE gene, and the DD allele is linked to increased ACE levels [66]. The relevance of the ACE/ACE2 balance in COVID-19 development and treatment has received much attention [66,67,68,69]. ACE and ACE2 balance the local vasodilator/antiproliferative and vasoconstrictor/proliferative activities of the RAS system [66]. Tissue damage, fibrosis, thrombosis, proliferation and inflammation may all be exacerbated if the ACE/ACE2 balance is disrupted. Thus, in contrast to ACE2, the ACE gene sequence may influence the results of the COVID-19 clinical trial [70,71]. Delanghe et al. found that the incidence of COVID-19 infections was adversely correlated with the frequency of the D allele in 25 European countries. Similar results were observed in 33 European, North African, and Middle Eastern nations, indicated that increasing D allele frequency was related with a decline in COVID-19 incidence but an increase in mortality [72]. In European and Asian nations, however, greater frequency of the I/I genotype was negatively associated with vulnerability to SARS-CoV-2 infection and subsequent death [73].

Similarly, an ecological analysis found that having a higher I/I genotype frequency was linked to lower COVID-19 mortality in 25 nations worldwide [74]. In Asian populations, numbers of the D allele were determined by the number of SARS-CoV-2 infected patients per million [75]. In Asian populations, mortality rates and the presence of the D allele revealed the substantial positive connection, demonstrating that greater levels of ACE are harmful to COVID-19 patients [75]. COVID-19 infection and fatality rates are both greater in the European population [73]. Since, the ACE DD genotype is more common in Europeans than in Asians, higher death rates in Europeans are likely to be accounted by ethnic variations in ACE I/D polymorphism allele prevalence.

#### 2.1.2. Angiotensin Converting Enzyme-2

Angiotensin-I and Angiotensin-II are cleaved into peptides such as Angiotensin 1–9 and Angiotensin 1–7, respectively, by ACE2, the master regulator of the RAS. These peptides are essential components in cardiovascular physiology control [76,77]. SARS-Cov-2 infection suppresses ACE2 expression and interferes with its homeostatic and defensive actions, causing inflammation [78]. ACE2 is present at various levels in most human tissues [79,80]. As SARS-CoV-2 attacks alveolar epithelial cells via the ACE2 receptor, the ACE2 expression levels in different organs might reveal genetic sensitivity to COVID-19 [81,82]. Single-cell RNA sequencing of various cell types in lung tissue revealed that bronchial branches contain a transient cell population with the ACE2 phenotype. Among these cells, 40% also express the transmembrane serine protease 2 (TMPRSS2) involved in the priming of the viral spike protein, thus making these cells more vulnerable to COVID-19 infection [83].

The ACE2 gene contains the regulatory elements for chromatin alteration and transcription factors that regulate expression levels epigenetically and hormonally. In several tissues, there is a link between ACE2 expression and age, gender, ethnicity, and BMI [84]. Asian females have the most significant connection with age, followed by sex and ethnic groupings. In a study of 305 people, the expression of the ACE2 gene in the nasal epithelium was explored, and it was discovered that younger children under the age of ten have the lowest ACE2 levels [85]. As a result, it was hypothesised that the reduced risk in children was linked to ACE2 expression levels that decreased with age [85]. Androgen receptor signaling governs the transcription of both ACE2 and TMPRSS2. Therefore, it is believed that androgen receptors regulate the production of ACE2 and TMPRSS2. This is the reason behind the gender differences in COVID-19 severity and the polymorphism in the androgen receptor linked to the condition [86]. ACE2 is substantially expressed in severe COVID-19 patients compared to controls, according to transcriptome analysis of 700 lung samples. More than one illness at the same time (co-morbidity) may increase the risk of serious COVID-19 [81]. On the other hand, other investigations have demonstrated a correlation between ACE2 expression and COVID-19 severity [84,87,88].

#### 2.1.3. Genetic Variants of ACE2

The genotype of ACE2 is linked to the binding affinity and structure of the protein, as well as serum concentration and systemic angiotensin levels [89]. In the NCBI database, about 18,000 single nucleotide variations of human ACE2 are denoted. From genetic susceptibility to COVID-19 infection, a comparative genomic investigation of ACE in different populations was conducted. Cao and his co-workers evaluated about 1700 ACE2 gene variations from different databases and the distribution system of expression quantitative trait loci (eQTLs) from the genotype and expression data of different tissues [90]. The investigating groups found various allele frequencies of ACE2 coding across the different populations (South Asian, East Asian, African, European, and mixed American populations). The allele frequencies of 11 of the 15 eQTLs that were linked to ACE2 expression were greater in East Asians (0.73–0.99) than in Europeans (0.44–0.65), which is indicative of the differential susceptibility to SARS-CoV-2 among different cultures [90]. The frequency of uncommon variations in the host genes encoding for virus entry machinery (ACE2, CtsB, CtsL, and TMPRSS2) are very variable among groups, suggesting that they might be crucial in SARS-CoV-2 entrance [91]. It was discovered that 13 genetic variations in ACE can improve the interaction among ACE2 and the viral S1 protein. The Europeans and Africans varied considerably with respect to rs73635825 (S19P), rs1244687367 (I21T), rs4646116 (K26R), rs781255386 (T27A), rs1199100713 (N64K), and rs142984500 (H378R) variations [91]. Various databases, and around 81000 human genomes, were used to look for ACE2 and TMPRSS2 polymorphisms [92]. The distribution of harmful mutations in ACE2 varied significantly between nine groups. African/African American (AFR) and Non-Finnish European (EUR) populations, for example, had 39% and 54% deleterious variations, respectively. The p.Met383Thr and p.Asp427Tyr variations was found in AFR groups, whereas the p.Pro389 his variation was found in Latino/Admixed American communities and was characterised as an inhibitory variant towards the interaction with the SARS-CoV-1 spike protein [92]. Gibson and his coworkers identified uncommon variations in distinct groups likely to impact spike protein binding [93]. They discovered that some exceptional variants are more common in certain groups or genders. For instance, the rs4646116 (p.Lys26Arg) allele is found at a greater frequency in Ashkenazi Jewish males than in EUR males; this allele was observed at higher frequencies in females. The difference in binding energy of the 15 ACE2 missense variations to SARS-CoV-2 indicates that Glu37Lys boosted binding with maximum efficiency, while Asn720Asp reduced it significantly. The N720 variation found near the TMPRSS2 cleavage site, is one of the most common mutations in Europe. The N720D variation alters the flexibility and stability of ACE2, and generates a preferred location for TMPRSS2 binding and cleavage, according to computational structural biology and molecular modelling [94]. As a result of the increased interaction between ACE2 and TMPRSS2, N720D carriers have higher S protein binding and viral entry [94]. 

Seventeen natural ACE2 coding variations were detected at the critical S protein binding sites in the natural ACE2 coding variants [95]. While most variants had the same binding affinity, the intermolecular interactions of rs143936283(E329G) and rs73635825(S19P) alleles were noticeably different. As a result, it is believed that the rs143936283 and rs73635825 alleles conferred resistance to SARS-CoV-2 attachment to the human ACE2 receptor [95]. K26R and I468V variations can impact the binding properties of S proteins by enhancing binding free-energy and lowering the binding affinity, according to molecular dynamic simulations [80]. Non-Finnish Europeans are more likely to have the K26R variation mutated, whereas East Asians are more likely to have the I468V variant mutated [80]. Whole exome sequencing (WES) data from 6930 healthy Italian persons were used to identify potential variations that affect protein stability [96]. Missense variations p.(Asn720Asp), p.(Lys26Arg), and p.(Gly211Arg) were prevalent and anticipated to interfere with the structure and stability of ACE2 protein, whereas p.(Pro389His) and p.(Leu351Val) were found to be uncommon and predicted to interfere with the binding to the viral spike protein. Moreover, it was observed that the control group had statistically significant higher allelic variability, indicating that genetic background may be responsible for the individual variations related to COVID-19 [96].

A large genomic dataset determined nine ACE2 variations anticipated to enhance susceptibility, and 17 projected to demonstrate reduced binding towards S protein and protection against SARS-CoV-2 transmission [97].

In contrast to these findings, a few studies claimed no link between ACE2 polymorphisms and illness severity [98]. WES investigated ACE2 genetic variations in 131 DNA samples from COVID-19 patients from a hospital in Italy, compared to a control group of 1000 people [99]. There was substantial variation in the frequency of the c.1888G>C p.(Asp630His) mutation across ethnically matched groups; nonetheless, there was no link seen between ACE2 variants and COVID-19 severity [99]. In silico simulations of ACE2-S1 protein binding kinetics have also shown some discrepancies [80,91,92,94,95,96]. The relevance of ACE2 genotypes in COVID-19 might be better understood with further functional investigations and genotype analysis. The SNPs variants detected for ACE and ACE2 shown in Table 2.

#### 2.1.4. Dipeptidyl Peptidase

Dipeptidyl Peptidase, also known as DPP4, is a critical human protein involved in numerous peptide interactions. This protein has a significant function in regulating diabetes [101]. However, it also interacts with several viral proteins [101]. A recent study showed that mutations in DPP4 were more evident in COVID-19 asymptomatic patients [46]. The severity of COVID-19 was also correlated with the down-regulation of DPP4 [102]. DPP4 expression was also associated with obesity, and subsequent investigations validated its association with COVID-19 [103,104,105,106]. In one study, missense and splice acceptor variants in DPP4 (c.95-2A > G, c.796G > A, c.1887 + 3G > A) were reported in COVID-19 patients and related to the severity of the disease [107].

#### 2.1.5. Furin

Cleavage is performed by furin, and a study determined that the cleavage site is critical for the entrance of the virus. The entry mechanism of SARS-CoV-2 into the host depends upon the cleavage of spike glycoprotein at a specific site [108]. Several mutations [15] were identified in the furin protein, and a few, such as R37C, R81C, R86Q, R637Q, R677W, R745Q, and S685P [7], showed a direct impact in lowering the risk of virus progression [109]. 

## 3. Genetic Resistance to Pathophysiological Changes Induced by Viruses 

Instead of inhibiting spread or transmission, plant breeders use genetic approaches to reduce pathological host responses towards infection, an approach known as “pathogen tolerance”. This incorporates immunological tolerance checkpoint processes that raise barriers to starting and maintaining innate and adaptive immune responses. The genetic resistance of coronavirus in bats could explain why they transmit these viruses asymptomatically at higher rates. The disease resistance established through tolerating microbe replication is evolutionarily more stable because it may be achieved without lowering the rate of microbial transmission and avoiding selection for resistance escape variants [110,111]. Since, tolerance mechanisms are forced to be genetically entrenched within the host species, they are also persistent. As even more people withstand the virus while transmitting it (asymptomatic shedders), infection rates among those who are genetically unable to sustain infection rise. Only tolerant hosts remain after these individuals die off. The displacement of ladybeetles by invading species asymptomatically transmits a more significant load of fungal infections due to genetically enhanced innate immune responses, which represent this type of “germ warfare” [112]. Contrary to popular belief, CD8+ T cell interactions to viruses may predominantly be the genetic basis for infection tolerance. More robust CD8+ T-cell responses to influenza epitopes do not appear to decrease viral transmission. These T cells might explain why there is less morbidity when a new influenza zoonosis has been established in the community [113,114]. The Epstein-Barr virus (EBV) causes lifetime infection in most individuals, which may be endured asymptomatically by having a large number of circulating CD8+ T cells fighting against the virus. Inheritance of a genetic mutation in SH2D1A, which produces the SLAM-associated protein, an intracellular adapter for SLAM-family receptors of CD8+ T cells, does not generate these CD8+ T-cell responses [115]. In people with SH2D1A deficiency, EBV causes morbid pathogenic immunological responses, culminating in severe mononucleosis and, in some instances, a lethal “cytokine storm” leading to hemophagocytic lymphohistiocytosis [116,117,118]. The relevance of addressing resistance to viral transmission and pathogenesis independently for SARS-CoV-2 is essential and significant. The initial batch of Salk vaccinations was ineffective against poliovirus infection and fecal–oral transmission. Nonetheless, it demonstrated protection against CNS viral infection and poliomyelitis [12,119]. This characteristic of decreasing index diagnosis but not virus transmission has resulted in silent epidemics of wild poliovirus [120]. The Sabin oral poliovirus vaccine, on the other hand, inhibits transmission and has become a milestone of poliovirus control, although it cannot be employed alone due to a unique reversion to the paralysis-inducing strain [119]. In experimental challenge studies, a non-replicating adenovirus vector encoding the SARS-CoV-2 spike protein evoked the virus-reactive CD8+ T cells and only lowered titers of neutralising antibodies in non-human primates. These titers were inadequate to protect the animals from viral respiratory tract disease but reduced propagation and pathology within the lung [121]. A comparable MERS vaccination did not protect camels from MERS coronavirus transmission [122]. This increases the prospect that first-generation SARS-CoV-2 vaccines may be a move forward in reducing COVID-19 hospitalisation rates but a step back in nurturing “silent” outbreaks within the unvaccinated communities and people who are unable to mount a CD8+ T-cell response.

## 4. Genetic Components for Immune Tolerance

### 4.1. Interferons

Interferons (IFN) are cytokines that, when secreted, activate numerous genes and further activate signal transduction pathways. The types of interferons known to date, namely Type I, Type II, and Type III, are involved in the induction of anti-viral host defence mechanisms [123]. Several studies have found that type I interferons are critical for controlling SARS-CoV-2 infection [124,125]. One of the genes induced by the IFN response is ACE2, which has been shown to be critical for viral entry into host cells. However, this induction is species-specific, as it is shown to occur in humans only, and not in mice. The activation of ACE2 acts to protect the lung during viral pathogenesis [126]. 

Type I interferons act by specific binding to interferon receptors (IFNARs) through an autocrine signaling process, resulting in copious amounts of IFN-α. There are two variants of IFNARs, IFNAR1 and IFNAR2. IFNRs act through Receptor Tyrosine Kinase 2 (TYK2) to induce phosphorylation of downstream target proteins [127]. The genetic variation near the locus coding for TYK (rs74956615) has been associated with disease severity in COVID-19 patients [49]. Lack of IFNARs results in the down-modulation of the anti-viral defence mechanism via reduced activity of IFN-α/β [128]. Variants of IFNAR1 (p.Trp73Cys, p.Ser422Arg, p.Pro335del) and IFNAR2 (p.Glu140fs) have been observed through genetic screening in severe COVID-19 infections [6]. The variations in the intron region of IFNRs are also linked to the severity of COVID-19 [49].

### 4.2. Interleukins

Polymorphism in the gene responsible for IL-6 production is associated with SARS-CoV-2 clearance, sustained anti-viral immune response and weakened adaptive immune functions [129,130,131]. In general, enhanced levels of IL-6 have been observed in patients with acute respiratory distress, thus highlighting the protective role of IL-6 in maintaining homeostasis within lung tissue [132]. 

In a recent study, five groups of patients, namely, mild, moderate, severe, critical, and asymptomatic, with varying degrees of COVID-19 severity, were studied for the presence of genetic variants. A total of 22.5 million gene variants were obtained from 332 COVID-19 patients [46]. From all the variants, the locus encoding for TMEM189-UBE2V1, a component of the IL-1 pathway, displayed maximum significance (SNP rs6020298) in terms of disease severity. 

### 4.3. Toll-Like Receptors

Toll-Like Receptors (TLRs) belong to an innate defence mechanism termed Pattern Recognition Receptors (PRRs). These PRRs recognise pathogen-associated molecular patterns (PAMPs), which are specific to each pathogen, to elicit a non-specific and specific immune response. Several families of receptors serve as the PRRs, of which TLRs are most abundant and are expressed on various host cells. TLR isoforms ranging from one to ten have been identified [133]. This family of receptors is involved in recognition of the double-stranded RNA or single-stranded RNA genome of the viruses. TLR7 and TLR8 are involved in sensing the ssRNA of viruses, which induces type I interferons and other pro-inflammatory cytokines. Interferon-activated transcription factors such as IRF3 and IRF7 play crucial roles in this signalling pathway [134]. Recently, the role of TLRs and IRFs was highlighted in the immune activation in severe COVID-19 infections. Eight genetic loci were linked to the severe infected cases, including TLR3, IRF3, IRF7, IFNAR1 and IFNAR2. A few cases showed genetic variations, namely, hrX(GRCh37):g.12905756_12905759del and ChrX(GRCh37):g.12906010G>T) led to the loss of function of TLR7 [135]. 

### 4.4. MHC

The Major Histocompatibility Complex (MHC) is required for the presentation of endogenous and exogenous antigens to Th and Tc cells. The genetic locus encoding for MHC is called the Human Leukocyte Antigen (HLA). This locus shows polymorphism, which accounts for the ability of MHC molecules to display the antigenic peptides from various pathogens encountered in hosts. The MHC polymorphism is also the reason behind immune response variations in people exposed to the same or similar infectious agents [136]. The HLA locus comprises about 240 genes and encodes for three MHC classes (I, II and III). The MHC classes I and II enable the immune response to differentiate between self and non-self-antigens. MHC molecules having enhanced specificity towards the antigenic peptides from SARS-CoV-2 result in better protection from virus-induced pathophysiological changes. A few reports have highlighted the relationship between HLA genetic loci and COVID-19 severity. It was observed that the frequency of occurrences of C (07:29) and B (15:27) alleles is much higher in COVID-19 patients [46]. Another study found that differences in HLA loci could result in differential induction of an anti-viral immune response mediated by T cells, which could impact disease severity. Computational studies revealed prospective alleles linked to COVID-19 severity, with HLA-B (46:01) having the lowest predicted binding sites towards the antigenic peptides derived from SARS-CoV-2 [137]. Other HLA alleles, such as B (15:03), display the full binding sites for SARS-CoV-2-derived antigenic peptides [138]. On the other hand, a few alleles, such as A (11:01), B (51:01), and C (14:02), if present, may lead to poor disease outcomes in severe COVID-19 cases [46]. 

### 4.5. Chemokines

Genome-wide association studies of severe COVID-19 patients have shown the relationship between the rs11386942 allele at locus 3-21.31 and the rs657152 allele at locus 9q34.2. The rs11386942 allele is linked to the reduced expression of chemokine receptor CXCR-6 with a concomitant increase in the expression of SLC6A20, a sodium transporter. SLC6A20 shares a functional interaction with the ACE2 receptor, which underlies its involvement in COVID-19 infection [139]. In another study, the proteomic profiling of three cohorts revealed the participation of CXCL-16 in COVID-19 disease. 

**Figure 2:**
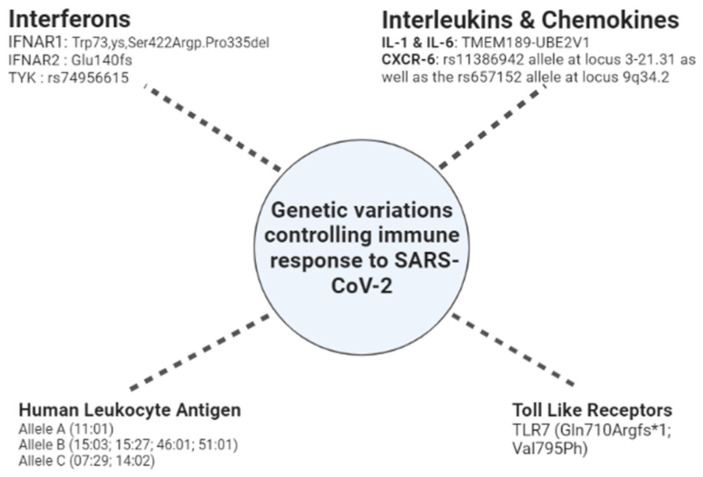
Genetic components responsible for immune response.

CXCL-16 and CXCR-6 represent a pair of chemokines and receptors encoded by genes within the 3p21.31 loci. This pair is also involved in generating and localising Tc memory cells to the infected airway cells [140,141]. A summary of the genetic components for immune tolerance is shown in Figure 2.

## 5. Cumulative Effect of Multiple SNPs 

A recent study was performed on a large set of SNPs, and found that 12.5 million SNPs did not participate in any pathway relevant to COVID-19; however, 27 SNPs showed significant resistance to infection. This study demonstrated that many routes may contribute to COVID-19 resistance. Consequently, the cumulative effect of SNPs can result in genetic resistance to COVID-19 [142]. 

## 6. Humoral Innate Immunity

Humoral immunity is governed by a large set of molecules that also act as antibodies; these molecules are called humoral fluid-phase pattern recognition molecules (PRMs). The association of these molecules with SARS-CoV2 was studied, and it was found that 13 of the PRMs investigated, including long pentraxin 3 (PTX3) and mannose-binding lectin (MBL), showed binding with viral protein [143]. Here, MBL was predicted to bind with Omicron variants. This study concluded that PRMs have a critical role in generating resistance against COVID-19 Another humoral immunity survey also suggested that COVID-19 infection severity is associated with superior immunity against the spike protein of the virus [144]. 

## 7. Susceptibility Prediction for COVID-19 Using Artificial Intelligence on Genetic Data

A study was conducted on 133 patients, and there were 381 known variants in a given set of genes. By deploying the gene variant data in an artificial neural network, a prediction model was built to establish the relation between these genetic variants and the severity of COVID-19. In this study, it was found that specific variations in five genes are critical for COVID-19 severity. The variation and the genes are as follows: rs2547438 (C3), rs2250656 (C3), rs1042580 (THBD), rs800292 (CFH), and rs414628 (CFHR1). The genetic data were complemented with the age and gender of the patients to improve the accuracy of the prediction model. This indicated the value of personalised medicine for COVID-19 treatment [145]. 

In another study, a set of six genes and their respective polymorphisms were examined to develop a machine learning model for predicting COVID-19 severity. In this investigation, MCP-1 of the GA genotype and G allele carriers were found to be considerably greater in severe COVID-19 patients than in asymptomatic COVID-19 patients [146]. The machine learning approach was also used to investigate human missense single nucleotide variants (SNVs) altering phosphorylation sites modulated by SARS-CoV-2 infection. This study indicated that phosphorylation sites could be altered in SARS-CoV-2 infection, which can alter kinase signalling. The single nucleotide variants (SNVs) detected at the phosphosites can be directly associated with the virus responses [147].

## 8. Conclusions

This review comprehensively describes host genetic factors that play a crucial role in resistance to SARS-CoV-2 infection and transmission. These factors have been identified by several genomic profiling studies in various populations with varying degrees of severity of COVID-19, including viral entry, pathophysiological changes, and immune responses. Some factors contribute towards resistance to virus entry, while others impact the immune response to the virus infection. Allelic variations at these gene loci influence disease outcome, thus affecting disease transmission within a population. Moreover, an AI-based prediction model can be used to determine COVID-19 susceptibility using genetic data.

## Figures and Tables

**Table 1:** Genetic factors influencing the susceptibility.

Sr. No.	Genetic Factor	Effect on Disease Outcome	Reference
1.	IFNAR1, IFNAR2, IRF3, IRF7	Impaired type I IFN signaling pathway, compromised protective immunity & poor control over SARS-CoV-2 infection	[28,29,30,31]
2.	TLR3 & TLR7	Neutralising antibodies against type I IFN resulting in virus spread	[29,32]
3.	ABO	Function as co-receptor for virus entry	[33,34]
4.	ACE/ACE2	Virus entry & tropism	[35,36,37,38,39]
5.	TMPRSS2	Priming & activation of Spike (S) protein	[40,41,42]
6.	TMEM41B; TMEM189-UBE2V1	Entry & early stage replication of the virus	[43,44,45,46]
7.	TYK2	Enhancement of disease severity	[47,48,49]
8.	HLA	Altered presentation of SARS-CoV-2 derived antigenic peptides to T_H_ cells.	[47]
9.	CXCL-16 & CXCR-6	Localisation of resident Tc memory cells in the infected airway	[50]

**Table 2:** SNPs detected in genetic locus encoding for ACE/ACE2.

Group	Cohort/Dataset	Population under Study	SNPs	Controlling Phenotype	Reference
Cao et al., 2020	ChinaMAP (China Metabolic Analytics Project) database 1KGP (1000 Genomes Project) database	South Asian, East Asian, African, European, and mixed American population	Lys26Arg, Ile468Val, Ala627Val, Asn638Ser, Ser692Pro, Asn720Asp, and Leu731Ile/Leu731Phe	Differential ACE2 expression and function	[90]
Darbani (2020)	13 ACE gene variations	Europeans, Africans, Asians, and Americans	S19P, I21T/V, E23K, A25T, K26R, T27A, E35D/K, FK, Y50F, N51D/S, M62V, N64K, K68E, F72V, E75G, M82I, T92I, Q102P, G220S, H239Q, G326E, E329G, G352V, D355N, H378R, Q388L, P389H, E467K, H505R, R514G/, and Y515C	Interaction among ACE2 and S1 protein- viral entry and infection	[91]
Hou et al., 2020	81,000 human genomes from the following databases: (i) Genome Aggregation Database (gnomAD v3: gnomad.broadinstitute.org, covering 9 geographical areas), (ii) Exome Sequencing Project (ESP: evs.gs.washington.edu/EVS/), and (iii) 1000 Genomes Project (1KGP, www.internationalgenome.org).	African/African American (AFR), Non-Finnish European (EUR) populations, Latino/Admixed American communities (LAM)	p.Met383Thr, p.Pro389His, and p.Asp427Tyr-	inhibits the interaction between ACE2 and the spike protein of SARS-CoV-2	[92]
Gibson et al. (2020)	gnomAD database of human genetic variation catalogues coding variants from 141,456 adults	Jewish and non-Finnish European males	p.Lys26Arg; N720D	increased interaction between ACE2 and TMPRSS2	[93]
Hussain et al., 2020	17 natural ACE2 allelic variations	NA	E329G and S19P	conferred resistance to SARS-CoV-2 attachment to the human ACE2 receptor	[95]
Benetti et al., 2020	Network of Italian Genomes (NIG) and Whole Genome Sequencing Data	Italian control population (*n* = 6930)	p.(Asn720Asp), p.(Lys26Arg), and p.(Gly211Arg)	interfere with ACE2 protein structure and stability	[96]
			p.(Pro389His) and p.(Leu351Val)	interfere with SARS-CoV-2 spike protein binding	
Li et al., 2020	gnomAD v2.1 exomes	Non-Finnish Europeans, East Asians (Asians and Caucasians)	K26R and I468V	impact binding properties to S protein	[80]
Ren et al., 2022	gnomAD	Global	K26E, E37K	Differential binding efficiency of SARS-CoV-2	[35]
Fawzy et al., 2022	PubMed, Web of Science, Scopus, and Cochrane CENTRAL	Global	Position 24-26 (QAK→KAE) Position 31 (K→D) Position 41 (Y→A) Position 68 (K→D) Position 82-84 (MYP→NFS) Position 169 (R→Q) Position 271 (W→Q) Position 273 (R→Q) Position 345 H→A Position 353 K→H, A/D Position 355 D→A Position 357 R→A Position 383 M→A Position 389 P→A Position 393 R→A Position 425-427 SPD→PSN Position 481 K→Q Position 505 H→A Position 514 R→Q Position 559 R→S	Changes in the enzymatic activity of ACE-2 and/or changed binding to spike protein	[100]

## Data Availability

Not applicable.

## References

[1] Bastard P., Rosen L.B., Zhang Q., Michailidis E., Hoffmann H.-H., Zhang Y., Dorgham K., Philippot Q., Rosain J., Béziat V. (2020). Autoantibodies against type I IFNs in patients with life-threatening COVID-19. Science.

[2] Zhang Q., Bastard P., Bolze A., Jouanguy E., Zhang S.-Y., Cobat A., Notarangelo L.D., Su H.C., Abel L., Casanova J.-L. (2020). Life-Threatening COVID-19: Defective Interferons Unleash Excessive Inflammation. Med.

[3] Cerami C., Popkin-Hall Z.R., Rapp T., Tompkins K., Zhang H., Muller M.S., Basham C., Whittelsey M., Chhetri S.B., Smith J. (2022). Household Transmission of Severe Acute Respiratory Syndrome Coronavirus 2 in the United States: Living Density, Viral Load, and Disproportionate Impact on Communities of Color. Clin. Infect. Dis..

[4] Madewell Z.J., Yang Y., Longini I.M., Halloran M.E., Dean N.E. (2020). Household Transmission of SARS-CoV-2: A Systematic Review and Meta-analysis. JAMA Netw. Open.

[5] Reukers D.F.M., van Boven M., Meijer A., Rots N., Reusken C., Roof I., van Gageldonk-Lafeber A.B., van der Hoek W., van den Hof S. (2022). High Infection Secondary Attack Rates of Severe Acute Respiratory Syndrome Coronavirus 2 in Dutch Households Revealed by Dense Sampling. Clin. Infect. Dis..

[6] Zhang Y., Qin L., Zhao Y., Zhang P., Xu B., Li K., Liang L., Zhang C., Dai Y., Feng Y. (2020). Interferon-Induced Transmembrane Protein 3 Genetic Variant rs12252-C Associated with Disease Severity in Coronavirus Disease 2019. J. Infect. Dis..

[7] Bastard P., Gervais A., Le Voyer T., Rosain J., Philippot Q., Manry J., Michailidis E., Hoffmann H.-H., Eto S., Garcia-Prat M. (2021). Autoantibodies neutralizing type I IFNs are present in ~4% of uninfected individuals over 70 years old and account for ~20% of COVID-19 deaths. Sci. Immunol..

[8] Asano T., Boisson B., Onodi F., Matuozzo D., Moncada-Velez M., Maglorius Renkilaraj M.R.L., Zhang P., Meertens L., Bolze A., Materna M. (2021). X-linked recessive TLR7 deficiency in ~1% of men under 60 years old with life-threatening COVID-19. Sci. Immunol..

[9] Galani I.-E., Rovina N., Lampropoulou V., Triantafyllia V., Manioudaki M., Pavlos E., Koukaki E., Fragkou P.C., Panou V., Rapti V. (2021). Untuned antiviral immunity in COVID-19 revealed by temporal type I/III interferon patterns and flu comparison. Nat. Immunol..

[10] Bastard P., Michailidis E., Hoffmann H.-H., Chbihi M., Le Voyer T., Rosain J., Philippot Q., Seeleuthner Y., Gervais A., Materna M. (2021). Auto-antibodies to type I IFNs can underlie adverse reactions to yellow fever live attenuated vaccine. J. Exp. Med..

[11] Shelton J.F., Shastri A.J., Ye C., Weldon C.H., Filshtein-Sonmez T., Coker D., Symons A., Esparza-Esparza-Gordillo J., Aslibekyan S., The 23andMe COVID-19 Team (2021). Trans-ancestry analysis reveals genetic and nongenetic associations with COVID-19 susceptibility and severity. Nat. Genet..

[12] COVID-19 Host Genetics Initiative (2021). Mapping the human genetic architecture of COVID-19. Nature.

[13] Zhang Y., Garner R., Salehi S., La Rocca M., Duncan D. (2021). Association between ABO blood types and coronavirus disease 2019 (COVID-19), genetic associations, and underlying molecular mechanisms: A literature review of 23 studies. Ann. Hematol..

[14] Freeman E.E., McMahon D.E., Lipoff J.B., Rosenbach M., Kovarik C., Takeshita J., French L.E., Thiers B.H., Hruza G.J., Fox L.P. (2020). Pernio-like skin lesions associated with COVID-19: A case series of 318 patients from 8 countries. J. Am. Acad. Derm..

[15] Tan S.W., Tam Y.C., Oh C.C. (2021). Skin manifestations of COVID-19: A worldwide review. JAAD Int..

[16] Crow Y.J., Manel N. (2015). Aicardi–Goutières syndrome and the type I interferonopathies. Nat. Rev. Immunol..

[17] Colmenero I., Santonja C., Alonso-Riaño M., Noguera-Morel L., Hernández-Martín A., Andina D., Wiesner T., Rodríguez-Peralto J.L., Requena L., Torrelo A. (2020). SARS-CoV-2 endothelial infection causes COVID-19 chilblains: Histopathological, immunohistochemical and ultrastructural study of seven paediatric cases. Br. J. Derm..

[18] Wei J., Alfajaro M.M., DeWeirdt P.C., Hanna R.E., Lu-Culligan W.J., Cai W.L., Strine M.S., Zhang S.-M., Graziano V.R., Schmitz C.O. (2021). Genome-wide CRISPR Screens Reveal Host Factors Critical for SARS-CoV-2 Infection. Cell.

[19] Daniloski Z., Jordan T.X., Wessels H.-H., Hoagland D.A., Kasela S., Legut M., Maniatis S., Mimitou E.P., Lu L., Geller E. (2021). Identification of Required Host Factors for SARS-CoV-2 Infection in Human Cells. Cell.

[20] Wang R., Simoneau C.R., Kulsuptrakul J., Bouhaddou M., Travisano K.A., Hayashi J.M., Carlson-Stevermer J., Zengel J.R., Richards C.M., Fozouni P. (2021). Genetic Screens Identify Host Factors for SARS-CoV-2 and Common Cold Coronaviruses. Cell.

[21] Hoffmann M., Kleine-Weber H., Schroeder S., Krüger N., Herrler T., Erichsen S., Schiergens T.S., Herrler G., Wu N.-H., Nitsche A. (2020). SARS-CoV-2 Cell Entry Depends on ACE2 and TMPRSS2 and Is Blocked by a Clinically Proven Protease Inhibitor. Cell.

[22] Horowitz J.E., Kosmicki J.A., Damask A., Sharma D., Roberts G.H.L., Justice A.E., Banerjee N., Coignet M.V., Yadav A., Leader J.B. (2020). Genome-Wide Analysis in 756,646 Individuals Provides First Genetic Evidence that ACE2 Expression Influences COVID-19 Risk and Yields Genetic Risk Scores Predictive of Severe Disease; Genetic and Genomic Medicine. http://medrxiv.org/lookup/doi/10.1101/2020.12.14.20248176.

[23] Suryamohan K., Diwanji D., Stawiski E.W., Gupta R., Miersch S., Liu J., Chen C., Jiang Y.-P., Fellouse F.A., Sathirapongsasuti J.F. (2021). Human ACE2 receptor polymorphisms and altered susceptibility to SARS-CoV-2. Commun. Biol..

[24] Schneider W.M., Luna J.M., Hoffmann H.-H., Sánchez-Rivera F.J., Leal A.A., Ashbrook A.W., Le Pen J., Ricardo-Lax I., Michailidis E., Peace A. (2021). Genome-Scale Identification of SARS-CoV-2 and Pan-coronavirus Host Factor Networks. Cell.

[25] Hoffmann H.-H., Schneider W.M., Rozen-Gagnon K., Miles L.A., Schuster F., Razooky B., Jacobson E., Wu X., Yi S., Rudin C.M. (2021). TMEM41B Is a Pan-flavivirus Host Factor. Cell.

[26] Gordon D.E., Jang G.M., Bouhaddou M., Xu J., Obernier K., White K.M., O’Meara M.J., Rezelj V.V., Guo J.Z., Swaney D.L. (2020). A SARS-CoV-2 protein interaction map reveals targets for drug repurposing. Nature.

[27] Hoffmann H.-H., Sánchez-Rivera F.J., Schneider W.M., Luna J.M., Soto-Feliciano Y.M., Ashbrook A.W., Le Pen J., Leal A.A., Ricardo-Lax I., Michailidis E. (2021). Functional interrogation of a SARS-CoV-2 host protein interactome identifies unique and shared coronavirus host factors. Cell Host Microbe.

[28] Zhang Q., Matuozzo D., Le Pen J., Lee D., Moens L., Asano T., Bohlen J., Liu Z., Moncada-Velez M., Kendir-Demirkol Y. (2022). Recessive inborn errors of type I IFN immunity in children with COVID-19 pneumonia. J. Exp. Med..

[29] Zhang Q., Bastard P., Liu Z., Le Pen J., Moncada-Velez M., Chen J., Ogishi M., Sabli I.K.D., Hodeib S., Korol C. (2020). Inborn errors of type I IFN immunity in patients with life-threatening COVID-19. Science.

[30] Gozman L., Perry K., Nikogosov D., Klabukov I., Shevlyakov A., Baranova A. (2021). A Role of Variance in Interferon Genes to Disease Severity in COVID-19 Patients. Front. Genet..

[31] Smieszek S.P., Polymeropoulos V.M., Xiao C., Polymeropoulos C.M., Polymeropoulos M.H. (2021). Loss-of-function mutations in IFNAR2 in COVID-19 severe infection susceptibility. J. Glob. Antimicrob. Resist..

[32] van der Made C.I., Simons A., Schuurs-Hoeijmakers J., van den Heuvel G., Mantere T., Kersten S., van Deuren R.C., Steehouwer M., van Reijmersdal S.V., Jaeger M. (2020). Presence of Genetic Variants Among Young Men with Severe COVID-19. JAMA.

[33] Bullerdiek J., Reisinger E., Rommel B., Dotzauer A. (2022). ABO Blood Groups and the Risk of SARS-CoV-2 Infection. Protoplasma.

[34] Ellinghaus D., Degenhardt F., Bujanda L., Buti M., Albillos A., Invernizzi P., Fernández J., Prati D., Baselli G., Asselta R. (2020). The ABO blood group locus and a chromosome 3 gene cluster associate with SARS-CoV-2 respiratory failure in an Italian-Spanish genome-wide association analysis. medRxiv.

[35] Ren W., Zhu Y., Lan J., Chen H., Wang Y., Shi H., Feng F., Chen D.-Y., Close B., Zhao X. (2022). Susceptibilities of Human ACE2 Genetic Variants in Coronavirus Infection. J. Virol..

[36] Yang Z., Macdonald-Dunlop E., Chen J., Zhai R., Li T., Richmond A., Klarić L., Pirastu N., Ning Z., Zheng C. (2022). Genetic Landscape of the ACE2 Coronavirus Receptor. Circulation.

[37] Chen F., Zhang Y., Li X., Li W., Liu X., Xue X. (2021). The Impact of ACE2 Polymorphisms on COVID-19 Disease: Susceptibility, Severity, and Therapy. Front. Cell. Infect. Microbiol..

[38] MacGowan S.A., Barton M.I., Kutuzov M., Dushek O., van der Merwe P.A., Barton G.J. (2022). Missense variants in human ACE2 strongly affect binding to SARS-CoV-2 Spike providing a mechanism for ACE2 mediated genetic risk in Covid-19: A case study in affinity predictions of interface variants. PLoS Comput. Biol..

[39] Wang B., Zhao J., Liu S., Feng J., Luo Y., He X., Wang Y., Ge F., Wang J., Ye B. (2022). ACE2 decoy receptor generated by high-throughput saturation mutagenesis efficiently neutralizes SARS-CoV-2 and its prevalent variants. Emerg. Microbes Infect..

[40] David A., Parkinson N., Peacock T.P., Pairo-Castineira E., Khanna T., Cobat A., Tenesa A., Sancho-Shimizu V., Casanova J.-L., Abel L. (2022). A common TMPRSS2 variant has a protective effect against severe COVID-19. Curr. Res. Transl. Med..

[41] Rokni M., Heidari Nia M., Sarhadi M., Mirinejad S., Sargazi S., Moudi M., Saravani R., Rahdar S., Kargar M. (2022). Association of TMPRSS2 Gene Polymorphisms with COVID-19 Severity and Mortality: A Case-Control Study with Computational Analyses. Appl. Biochem. Biotechnol..

[42] Duman N., Tuncel G., Bisgin A., Bozdogan S.T., Sag S.O., Gul S., Kiraz A., Balta B., Erdogan M., Uyanik B. (2022). Analysis of ACE2 and TMPRSS2 coding variants as a risk factor for SARS-CoV-2 from 946 whole-exome sequencing data in the Turkish population. J. Med. Virol..

[43] Sun L., Zhao C., Fu Z., Fu Y., Su Z., Li Y., Zhou Y., Tan Y., Li J., Xiang Y. (2021). Genome-scale CRISPR screen identifies TMEM41B as a multi-function host factor required for coronavirus replication. PLoS Pathog..

[44] Trimarco J.D., Heaton B.E., Chaparian R.R., Burke K.N., Binder R.A., Gray G.C., Smith C.M., Menachery V.D., Heaton N.S. (2021). TMEM41B is a host factor required for the replication of diverse coronaviruses including SARS-CoV-2. PLoS Pathog..

[45] Hama Y., Morishita H., Mizushima N. (2022). Regulation of ER-derived membrane dynamics by the DedA domain-containing proteins VMP1 and TMEM41B. EMBO Rep..

[46] Wang F., Huang S., Gao R., Zhou Y., Lai C., Li Z., Xian W., Qian X., Li Z., Huang Y. (2020). Initial whole-genome sequencing and analysis of the host genetic contribution to COVID-19 severity and susceptibility. Cell Discov..

[47] Velavan T.P., Pallerla S.R., Rüter J., Augustin Y., Kremsner P.G., Krishna S., Meyer C.G. (2021). Host genetic factors determining COVID-19 susceptibility and severity. eBioMedicine.

[48] Verma A., Tsao N.L., Thomann L.O., Ho Y.-L., Iyengar S.K., Luoh S.-W., Carr R., Crawford D.C., Efird J.T., Huffman J.E. (2022). A Phenome-Wide Association Study of genes associated with COVID-19 severity reveals shared genetics with complex diseases in the Million Veteran Program. PLoS Genet..

[49] Pairo-Castineira E., Clohisey S., Klaric L., Bretherick A.D., Rawlik K., Pasko D., Walker S., Parkinson N., Fourman M.H., Russell C.D. (2021). Genetic mechanisms of critical illness in COVID-19. Nature.

[50] Smieszek S.P., Polymeropoulos V.M., Polymeropoulos C.M., Przychodzen B.P., Birznieks G., Polymeropoulos M.H. (2022). Elevated plasma levels of CXCL16 in severe COVID-19 patients. Cytokine.

[51] Jlizi A., Edouard J., Fadhlaoui-Zid K., Frigi S., Debré P., Slim A., Theodorou I., El Gaaied A.B.A., Carpentier W. (2007). Identification of the CCR5-Δ32 HIV resistance allele and new mutations of the CCR5 gene in different Tunisian populations. Hum. Immunol..

[52] Khanaliha K., Bokharaei-Salim F., Donyavi T., Nahand J.S., Marjani A., Jamshidi S., Khatami A., Moghaddas M., Esghaei M., Fakhim A. (2022). Evaluation of CCR5-Δ32 mutation and HIV-1 surveillance drug-resistance mutations in peripheral blood mononuclear cells of long-term non progressors of HIV-1-infected individuals. Future Virol..

[53] Fath-Elrahman M.H., Alkarsany M., Nour B.Y.M., Abakar A.D., Mhammed A.E., Elzaki S.G., Osman E., Elshafia M., Ahmed E.A. (2022). Rating of CCR5-Delta 32 Homozygous Mutation in Sudanese HIV Patients and Sex Workers. WJA.

[54] Veerabathiran R., Mansoor S.A., Kalarani I.B., Mohammed V. (2022). Gene-editing of CCR5 for the Treatment of HIV: A Novel Therapeutic Approach. TJI.

[55] Abel L., Fellay J., Haas D.W., Schurr E., Srikrishna G., Urbanowski M., Chaturvedi N., Srinivasan S., Johnson D.H., Bishai W.R. (2018). Genetics of human susceptibility to active and latent tuberculosis: Present knowledge and future perspectives. Lancet Infect. Dis..

[56] Boisson-Dupuis S. (2020). The monogenic basis of human tuberculosis. Hum. Genet..

[57] Altare F., Ensser A., Breiman A., Reichenbach J., Baghdadi J.E., Fischer A., Emile J., Gaillard J., Meinl E., Casanova J. (2001). Interleukin-12 Receptor β1 Deficiency in a Patient with Abdominal Tuberculosis. J. Infect. Dis..

[58] Boisson-Dupuis S., El Baghdadi J., Parvaneh N., Bousfiha A., Bustamante J., Feinberg J., Samarina A., Grant A.V., Janniere L., El Hafidi N. (2011). IL-12Rβ1 Deficiency in Two of Fifty Children with Severe Tuberculosis from Iran, Morocco, and Turkey. PLoS ONE.

[59] Kreins A.Y., Ciancanelli M.J., Okada S., Kong X.-F., Ramírez-Alejo N., Kilic S.S., El Baghdadi J., Nonoyama S., Mahdaviani S.A., Ailal F. (2015). Human TYK2 deficiency: Mycobacterial and viral infections without hyper-IgE syndrome. J. Exp. Med..

[60] Boisson-Dupuis S., Ramirez-Alejo N., Li Z., Patin E., Rao G., Kerner G., Lim C.K., Krementsov D.N., Hernandez N., Ma C.S. (2018). Tuberculosis and impaired IL-23–dependent IFN-γ immunity in humans homozygous for a common *TYK2* missense variant. Sci. Immunol..

[61] Kerner G., Laval G., Patin E., Boisson-Dupuis S., Abel L., Casanova J.-L., Quintana-Murci L. (2021). Human ancient DNA analyses reveal the high burden of tuberculosis in Europeans over the last 2,000 years. Am. J. Hum. Genet..

[62] de Prost N., Bastard P., Arrestier R., Fourati S., Mahévas M., Burrel S., Dorgham K., Gorochov G., Tandjaoui-Lambiotte Y., Azzaoui I. (2021). Plasma Exchange to Rescue Patients with Autoantibodies Against Type I Interferons and Life-Threatening COVID-19 Pneumonia. J. Clin. Immunol..

[63] Koning R., Bastard P., Casanova J.L., Brouwer M.C., van de Beek D., with the Amsterdam U.M.C. (2021). COVID-19 Biobank Investigator. Autoantibodies against type I interferons are associated with multi-organ failure in COVID-19 patients. Intensive Care Med..

[64] Troya J., Bastard P., Planas-Serra L., Ryan P., Ruiz M., de Carranza M., Torres J., Martínez A., Abel L., Casanova J.-L. (2021). Neutralizing Autoantibodies to Type I IFNs in >10% of Patients with Severe COVID-19 Pneumonia Hospitalized in Madrid, Spain. J. Clin. Immunol..

[65] Ghafouri-Fard S., Noroozi R., Vafaee R., Branicki W., Poṡpiech E., Pyrc K., Łabaj P.P., Omrani M.D., Taheri M., Sanak M. (2020). Effects of host genetic variations on response to, susceptibility and severity of respiratory infections. Biomed. Pharmacother..

[66] Gemmati D., Bramanti B., Serino M.L., Secchiero P., Zauli G., Tisato V. (2020). COVID-19 and Individual Genetic Susceptibility/Receptivity: Role of ACE1/ACE2 Genes, Immunity, Inflammation and Coagulation. Might the Double X-Chromosome in Females Be Protective against SARS-CoV-2 Compared to the Single X-Chromosome in Males?. IJMS.

[67] Sriram K., Insel P.A. (2020). A hypothesis for pathobiology and treatment of COVID-19: The centrality of ACE1/ACE2 imbalance. Br. J. Pharm..

[68] Tseng Y., Yang R., Lu T. (2020). Two hits to the renin-angiotensin system may play a key role in severe COVID-19. Kaohsiung J. Med. Sci..

[69] Zamai L. (2020). The Yin and Yang of ACE/ACE2 Pathways: The Rationale for the Use of Renin-Angiotensin System Inhibitors in COVID-19 Patients. Cells.

[70] Zheng H., Cao J.J. (2020). Angiotensin-Converting Enzyme Gene Polymorphism and Severe Lung Injury in Patients with Coronavirus Disease 2019. Am. J. Pathol..

[71] Delanghe J.R., Speeckaert M.M., De Buyzere M.L. (2020). The host’s angiotensin-converting enzyme polymorphism may explain epidemiological findings in COVID-19 infections. Clin. Chim. Acta.

[72] Delanghe J.R., Speeckaert M.M., De Buyzere M.L. (2020). COVID-19 infections are also affected by human ACE1 D/I polymorphism. Clin. Chem. Lab. Med. (CCLM).

[73] Yamamoto N., Ariumi Y., Nishida N., Yamamoto R., Bauer G., Gojobori T., Shimotohno K., Mizokami M. (2020). SARS-CoV-2 infections and COVID-19 mortalities strongly correlate with ACE1 I/D genotype. Gene.

[74] Aung A.K., Aitken T., Teh B.M., Yu C., Ofori-Asenso R., Chin K.L., Liew D. (2020). Angiotensin converting enzyme genotypes and mortality from COVID-19: An ecological study. J. Infect..

[75] Pati A., Mahto H., Padhi S., Panda A.K. (2020). ACE deletion allele is associated with susceptibility to SARS-CoV-2 infection and mortality rate: An epidemiological study in the Asian population. Clin. Chim. Acta.

[76] Donoghue M., Hsieh F., Baronas E., Godbout K., Gosselin M., Stagliano N., Donovan M., Woolf B., Robison K., Jeyaseelan R. (2000). A Novel Angiotensin-Converting Enzyme–Related Carboxypeptidase (ACE2) Converts Angiotensin I to Angiotensin 1-9. Circ. Res..

[77] Gheblawi M., Wang K., Viveiros A., Nguyen Q., Zhong J.-C., Turner A.J., Raizada M.K., Grant M.B., Oudit G.Y. (2020). Angiotensin-Converting Enzyme 2: SARS-CoV-2 Receptor and Regulator of the Renin-Angiotensin System: Celebrating the 20th Anniversary of the Discovery of ACE2. Circ. Res..

[78] Verdecchia P., Cavallini C., Spanevello A., Angeli F. (2020). The pivotal link between ACE2 deficiency and SARS-CoV-2 infection. Eur. J. Intern. Med..

[79] Fam B.S.O., Vargas-Pinilla P., Amorim C.E.G., Sortica V.A., Bortolini M.C. (2020). ACE2 diversity in placental mammals reveals the evolutionary strategy of SARS-CoV-2. Genet. Mol. Biol..

[80] Li M.-Y., Li L., Zhang Y., Wang X.-S. (2020). Expression of the SARS-CoV-2 cell receptor gene ACE2 in a wide variety of human tissues. Infect. Dis. Poverty.

[81] Pinto B.G.G., Oliveira A.E.R., Singh Y., Jimenez L., Gonçalves A.N.A., Ogava R.L.T., Creighton R., Schatzmann Peron J.P., Nakaya H.I. (2020). ACE2 Expression Is Increased in the Lungs of Patients with Comorbidities Associated with Severe COVID-19. J. Infect. Dis..

[82] Zou X., Chen K., Zou J., Han P., Hao J., Han Z. (2020). Single-cell RNA-seq data analysis on the receptor ACE2 expression reveals the potential risk of different human organs vulnerable to 2019-nCoV infection. Front. Med..

[83] Lukassen S., Chua R.L., Trefzer T., Kahn N.C., Schneider M.A., Muley T., Winter H., Meister M., Veith C., Boots A.W. (2020). SARS-CoV-2 receptor ACE 2 and TMPRSS 2 are primarily expressed in bronchial transient secretory cells. EMBO J..

[84] Chen L., Li X., Chen M., Feng Y., Xiong C. (2020). The ACE2 expression in human heart indicates new potential mechanism of heart injury among patients infected with SARS-CoV-2. Cardiovasc. Res..

[85] Bunyavanich S., Do A., Vicencio A. (2020). Nasal Gene Expression of Angiotensin-Converting Enzyme 2 in Children and Adults. JAMA.

[86] McCoy J., Wambier C.G., Vano-Galvan S., Shapiro J., Sinclair R., Ramos P.M., Washenik K., Andrade M., Herrera S., Goren A. (2020). Racial variations in COVID-19 deaths may be due to androgen receptor genetic variants associated with prostate cancer and androgenetic alopecia. Are anti-androgens a potential treatment for COVID-19?. J. Cosmet. Derm..

[87] Cheng Y., Luo R., Wang K., Zhang M., Wang Z., Dong L., Li J., Yao Y., Ge S., Xu G. (2020). Kidney impairment is associated with in-hospital death of COVID-19 patients. medRxiv.

[88] El Baba R., Herbein G. (2020). Management of epigenomic networks entailed in coronavirus infections and COVID-19. Clin. Epigenet..

[89] Chen Y.Y., Zhang P., Zhou X.M., Liu D., Zhong J.C., Zhang C.J., Jin L.J., Yu H.M. (2018). Relationship between genetic variants of ACE 2 gene and circulating levels of ACE 2 and its metabolites. J. Clin. Pharm. Ther..

[90] Cao Y., Li L., Feng Z., Wan S., Huang P., Sun X., Wen F., Huang X., Ning G., Wang W. (2020). Comparative genetic analysis of the novel coronavirus (2019-nCoV/SARS-CoV-2) receptor ACE2 in different populations. Cell Discov..

[91] Darbani B. (2020). The Expression and Polymorphism of Entry Machinery for COVID-19 in Human: Juxtaposing Population Groups, Gender, and Different Tissues. IJERPH.

[92] Hou Y., Zhao J., Martin W., Kallianpur A., Chung M.K., Jehi L., Sharifi N., Erzurum S., Eng C., Cheng F. (2020). New insights into genetic susceptibility of COVID-19: An ACE2 and TMPRSS2 polymorphism analysis. BMC Med..

[93] Gibson W.T., Evans D.M., An J., Jones S.J. (2020). ACE 2 Coding Variants: A Potential X-linked Risk Factor for COVID-19 Disease. BioRxiv.

[94] Mohammad A., Marafie S.K., Alshawaf E., Abu-Farha M., Abubaker J., Al-Mulla F. (2020). Structural analysis of ACE2 variant N720D demonstrates a higher binding affinity to TMPRSS2. Life Sci..

[95] Hussain M., Jabeen N., Raza F., Shabbir S., Baig A.A., Amanullah A., Aziz B. (2020). Structural variations in human ACE2 may influence its binding with SARS-CoV-2 spike protein. J. Med. Virol..

[96] Benetti E., Tita R., Spiga O., Ciolfi A., Birolo G., Bruselles A., Doddato G., Giliberti A., Marconi C., Musacchia F. (2020). ACE2 gene variants may underlie interindividual variability and susceptibility to COVID-19 in the Italian population. Eur. J. Hum. Genet..

[97] Stawiski E.W., Diwanji D., Suryamohan K., Gupta R., Fellouse F.A., Sathirapongsasuti J.F., Liu J., Jiang Y.-P., Ratan A., Mis M. (2020). Human ACE2 receptor polymorphisms predict SARS-CoV-2 susceptibility. BioRxiv.

[98] Torre-Fuentes L., Matías-Guiu J., Hernández-Lorenzo L., Montero-Escribano P., Pytel V., Porta-Etessam J., Gómez-Pinedo U., Matías-Guiu J.A. (2021). *ACE2, TMPRSS2*, and Furin variants and SARS-CoV-2 infection in Madrid, Spain. J. Med. Virol..

[99] Novelli A., Biancolella M., Borgiani P., Cocciadiferro D., Colona V.L., D’Apice M.R., Rogliani P., Zaffina S., Leonardis F., Campana A. (2020). Analysis of ACE2 genetic variants in 131 Italian SARS-CoV-2-positive patients. Hum. Genom..

[100] Fawzy M.S., Ashour H., Shafie A.A.A., Dahman N.B.H., Fares A.M., Antar S., Elnoby A.S., Fouad F.M. (2022). The role of angiotensin-converting enzyme 2 (ACE2) genetic variations in COVID-19 infection: A literature review. Egypt J. Med. Hum. Genet..

[101] Deacon C.F. (2019). Physiology and Pharmacology of DPP-4 in Glucose Homeostasis and the Treatment of Type 2 Diabetes. Front. Endocrinol..

[102] Posadas-Sánchez R., Sánchez-Muñoz F., Guzmán-Martín C.A., Hernández-Díaz Couder A., Rojas-Velasco G., Fragoso J.M., Vargas-Alarcón G. (2021). Dipeptidylpeptidase-4 levels and DPP4 gene polymorphisms in patients with COVID-19. Association with disease and with severity. Life Sci..

[103] Kirino Y., Sei M., Kawazoe K., Minakuchi K., Sato Y. (2012). Plasma dipeptidyl peptidase 4 activity correlates with body mass index and the plasma adiponectin concentration in healthy young people. Endocr. J..

[104] Stengel A., Goebel-Stengel M., Teuffel P., Hofmann T., Buße P., Kobelt P., Rose M., Klapp B.F. (2014). Obese patients have higher circulating protein levels of dipeptidyl peptidase IV. Peptides.

[105] Ghorpade D.S., Ozcan L., Zheng Z., Nicoloro S.M., Shen Y., Chen E., Blüher M., Czech M.P., Tabas I. (2018). Hepatocyte-secreted DPP4 in obesity promotes adipose inflammation and insulin resistance. Nature.

[106] Simonnet A., Chetboun M., Poissy J., Raverdy V., Noulette J., Duhamel A., Labreuche J., Mathieu D., Pattou F., Jourdain M. (2020). High Prevalence of Obesity in Severe Acute Respiratory Syndrome Coronavirus-2 (SARS-CoV-2) Requiring Invasive Mechanical Ventilation. Obesity.

[107] Latini A., Agolini E., Novelli A., Borgiani P., Giannini R., Gravina P., Smarrazzo A., Dauri M., Andreoni M., Rogliani P. (2020). COVID-19 and Genetic Variants of Protein Involved in the SARS-CoV-2 Entry into the Host Cells. Genes.

[108] Peacock T.P., Goldhill D.H., Zhou J., Baillon L., Frise R., Swann O.C., Kugathasan R., Penn R., Brown J.C., Sanchez-David R.Y. (2021). The furin cleavage site in the SARS-CoV-2 spike protein is required for transmission in ferrets. Nat. Microbiol..

[109] Delshad M., Sanaei M.-J., Pourbagheri-Sigaroodi A., Bashash D. (2022). Host genetic diversity and genetic variations of SARS-CoV-2 in COVID-19 pathogenesis and the effectiveness of vaccination. Int. Immunopharmacol..

[110] Caldwell R.M., Schafer J.F., Compton L.E., Patterson F.L. (1958). Tolerance to Cereal Leaf Rusts. Science.

[111] Roy B.A., Kirchner J.W. (2000). Evolutionary dynamics of pathogen resistance and tolerance. Evolution.

[112] Vilcinskas A., Stoecker K., Schmidtberg H., Röhrich C.R., Vogel H. (2013). Invasive Harlequin Ladybird Carries Biological Weapons Against Native Competitors. Science.

[113] Karupiah G., Buller R.M., Van Rooijen N., Duarte C.J., Chen J. (1996). Different roles for CD4+ and CD8+ T lymphocytes and macrophage subsets in the control of a generalized virus infection. J. Virol..

[114] Panchanathan V., Chaudhri G., Karupiah G. (2006). Protective Immunity against Secondary Poxvirus Infection Is Dependent on Antibody but Not on CD4 or CD8 T-Cell Function. J. Virol..

[115] Detre C., Keszei M., Romero X., Tsokos G.C., Terhorst C. (2010). SLAM family receptors and the SLAM-associated protein (SAP) modulate T cell functions. Semin. Immunopathol..

[116] Chaganti S., Ma C.S., Bell A.I., Croom-Carter D., Hislop A.D., Tangye S.G., Rickinson A.B. (2008). Epstein-Barr virus persistence in the absence of conventional memory B cells: IgM+IgD+CD27+ B cells harbor the virus in X-linked lymphoproliferative disease patients. Blood.

[117] Dupré L., Andolfi G., Tangye S.G., Clementi R., Locatelli F., Aricò M., Aiuti A., Roncarolo M.-G. (2005). SAP controls the cytolytic activity of CD8+ T cells against EBV-infected cells. Blood.

[118] Palendira U., Low C., Bell A.I., Ma C.S., Abbott R.J.M., Phan T.G., Riminton D.S., Choo S., Smart J.M., Lougaris V. (2012). Expansion of somatically reverted memory CD8+ T cells in patients with X-linked lymphoproliferative disease caused by selective pressure from Epstein-Barr virus. J. Exp. Med..

[119] Hird T.R., Grassly N.C. (2012). Systematic Review of Mucosal Immunity Induced by Oral and Inactivated Poliovirus Vaccines against Virus Shedding following Oral Poliovirus Challenge. PLoS Pathog..

[120] Moran-Gilad J., Kaliner E., Gdalevich M., Grotto I. (2016). Public health response to the silent reintroduction of wild poliovirus to Israel, 2013–2014. Clin. Microbiol. Infect..

[121] van Doremalen N., Lambe T., Spencer A., Belij-Rammerstorfer S., Purushotham J.N., Port J.R., Avanzato V.A., Bushmaker T., Flaxman A., Ulaszewska M. (2020). ChAdOx1 nCoV-19 vaccine prevents SARS-CoV-2 pneumonia in rhesus macaques. Nature.

[122] Alharbi N.K., Qasim I., Almasoud A., Aljami H.A., Alenazi M.W., Alhafufi A., Aldibasi O.S., Hashem A.M., Kasem S., Albrahim R. (2019). Humoral Immunogenicity and Efficacy of a Single Dose of ChAdOx1 MERS Vaccine Candidate in Dromedary Camels. Sci. Rep..

[123] Acosta P.L., Byrne A.B., Hijano D.R., Talarico L.B. (2020). Human Type I Interferon Antiviral Effects in Respiratory and Reemerging Viral Infections. J. Immunol. Res..

[124] Channappanavar R., Fehr A.R., Vijay R., Mack M., Zhao J., Meyerholz D.K., Perlman S. (2016). Dysregulated Type I Interferon and Inflammatory Monocyte-Macrophage Responses Cause Lethal Pneumonia in SARS-CoV-Infected Mice. Cell Host Microbe.

[125] Channappanavar R., Perlman S. (2017). Pathogenic human coronavirus infections: Causes and consequences of cytokine storm and immunopathology. Semin. Immunopathol..

[126] Ziegler C.G.K., Allon S.J., Nyquist S.K., Mbano I.M., Miao V.N., Tzouanas C.N., Cao Y., Yousif A.S., Bals J., Hauser B.M. (2020). SARS-CoV-2 Receptor ACE2 Is an Interferon-Stimulated Gene in Human Airway Epithelial Cells and Is Detected in Specific Cell Subsets across Tissues. Cell.

[127] Su Q., Wang S., Baltzis D., Qu L., Raven J.F., Li S., Wong A.H., Koromilas A.E. (2007). Interferons induce tyrosine phosphorylation of the eIF2α kinase PKR through activation of Jak1 and Tyk2. EMBO Rep..

[128] Duncan C.J.A., Mohamad S.M.B., Young D.F., Skelton A.J., Leahy T.R., Munday D.C., Butler K.M., Morfopoulou S., Brown J.R., Hubank M. (2015). Human IFNAR2 deficiency: Lessons for antiviral immunity. Sci. Transl. Med..

[129] Bogdanović Z., Marinović-Terzić I., Kuret S., Jerončić A., Bradarić N., Forempoher G., Polašek O., Anđelinović Š., Terzić J. (2016). The impact of IL-6 and IL-28B gene polymorphisms on treatment outcome of chronic hepatitis C infection among intravenous drug users in Croatia. PeerJ.

[130] Barrett S. (2001). The natural course of hepatitis C virus infection after 22 years in a unique homogenous cohort: Spontaneous viral clearance and chronic HCV infection. Gut.

[131] Nattermann J., Vogel M., Berg T., Danta M., Axel B., Mayr C., Bruno R., Tural C., Klausen G., Clotet B. (2007). Effect of the interleukin-6 C174G gene polymorphism on treatment of acute and chronic hepatitis C in human immunodeficiency virus coinfected patients. Hepatology.

[132] Leisman D.E., Ronner L., Pinotti R., Taylor M.D., Sinha P., Calfee C.S., Hirayama A.V., Mastroiani F., Turtle C.J., Harhay M.O. (2020). Cytokine elevation in severe and critical COVID-19: A rapid systematic review, meta-analysis, and comparison with other inflammatory syndromes. Lancet Respir. Med..

[133] Takeda K., Kaisho T., Akira S. (2003). Toll-Like Receptors. Annu. Rev. Immunol..

[134] Lim K.-H., Staudt L.M. (2013). Toll-Like Receptor Signaling. Cold Spring Harb. Perspect. Biol..

[135] Amodio G., Gregori S. (2020). HLA-G genotype/expression/disease association studies: Success, hurdles, and perspectives. Front. Immunol.

[136] Dendrou C.A., Petersen J., Rossjohn J., Fugger L. (2018). HLA variation and disease. Nat. Rev. Immunol..

[137] Lin M., Tseng H.-K., Trejaut J.A., Lee H.-L., Loo J.-H., Chu C.-C., Chen P.-J., Su Y.-W., Lim K.H., Tsai Z.-U. (2003). Association of HLA class I with severe acute respiratory syndrome coronavirus infection. BMC Med. Genet..

[138] Nguyen A., David J.K., Maden S.K., Wood M.A., Weeder B.R., Nellore A., Thompson R.F. (2020). Human Leukocyte Antigen Susceptibility Map for Severe Acute Respiratory Syndrome Coronavirus 2. J. Virol..

[139] Vuille-dit-Bille R.N., Camargo S.M., Emmenegger L., Sasse T., Kummer E., Jando J., Hamie Q.M., Meier C.F., Hunziker S., Forras-Kaufmann Z. (2015). Human intestine luminal ACE2 and amino acid transporter expression increased by ACE-inhibitors. Amino Acids.

[140] Wein A.N., McMaster S.R., Takamura S., Dunbar P.R., Cartwright E.K., Hayward S.L., McManus D.T., Shimaoka T., Ueha S., Tsukui T. (2019). CXCR6 regulates localization of tissue-resident memory CD8 T cells to the airways. J. Exp. Med..

[141] Tu G., Ju M., Zheng Y., Hao G., Ma G., Hou J., Zhang X., Luo Z., Lu L. (2019). CXCL16/CXCR6 is involved in LPS-induced acute lung injury via P38 signalling. J. Cell Mol. Med..

[142] Szyda J., Dobosz P., Stojak J., Sypniewski M., Suchocki T., Kotlarz K., Mroczek M., Stępień M., Słomian D., Butkiewicz S. (2022). Beyond GWAS—Could Genetic Differentiation within the Allograft Rejection Pathway Shape Natural Immunity to COVID-19?. IJMS.

[143] Stravalaci M., Pagani I., Paraboschi E.M., Pedotti M., Doni A., Scavello F., Mapelli S.N., Sironi M., Perucchini C., Varani L. (2022). Recognition and inhibition of SARS-CoV-2 by humoral innate immunity pattern recognition molecules. Nat. Immunol..

[144] Guthmiller J.J., Stovicek O., Wang J., Changrob S., Li L., Halfmann P., Zheng N.-Y., Utset H., Stamper C.T., Dugan H.L. (2021). SARS-CoV-2 Infection Severity Is Linked to Superior Humoral Immunity against the Spike. mBio.

[145] Asteris P.G., Gavriilaki E., Touloumenidou T., Koravou E., Koutra M., Papayanni P.G., Pouleres A., Karali V., Lemonis M.E., Mamou A. (2022). Genetic prediction of ICU hospitalization and mortality in COVID-19 patients using artificial neural networks. J. Cell. Mol. Med..

[146] Dogan S., Mart Komurcu S.Z., Korkmaz M.D., Kaya E., Yavas S., Dogan S., Senturk Ciftci H., Dasdemir S. (2022). Effect of Chemokine Gene Variants on Covid-19 Disease Severity. Immunol. Investig..

[147] Pellegrina D., Bahcheli A.T., Krassowski M., Reimand J. (2022). Human phospho-signaling networks of SARS-CoV-2 infection are rewired by population genetic variants. Mol. Syst. Biol..

